# Differences in the soluble and insoluble proteome between primary tauopathies

**DOI:** 10.1002/alz.70401

**Published:** 2025-06-22

**Authors:** Tomas Kavanagh, Kaleah Balcomb, Stephanie Trgovcevic, Laura Nementzik, Evgeny Kanshin, Glenda Halliday, Beatrix Ueberheide, Eleanor Drummond

**Affiliations:** ^1^ Brain and Mind Centre and School of Medical Sciences University of Sydney Camperdown New South Wales Australia; ^2^ Proteomics Laboratory Division of Advanced Research Technologies and Department of Biochemistry and Molecular Pharmacology New York University Grossman School of Medicine New York New York USA; ^3^ Department of Biochemistry and Molecular Pharmacology New York University Grossman School of Medicine New York New York USA; ^4^ Department of Neurology New York University Grossman School of Medicine New York New York USA

**Keywords:** brain, corticobasal degeneration, human, insoluble, mass spectrometry, Pick's disease, progressive supranuclear palsy, proteomics, sarkosyl, sortilin, tau, tauopathy

## Abstract

**INTRODUCTION:**

Primary tauopathies, including corticobasal degeneration (CBD), Pick's disease (PiD), and progressive supranuclear palsy (PSP), have aggregated tau pathology in the brain. Many other proteins are likely altered in disease; however, these have not been well characterized.

**METHODS:**

We performed sarkosyl fractionation of *post mortem* human brain tissue to enrich soluble and insoluble proteins from CBD, PiD, and PSP cases (*n* = 5/group). We assessed differences in the soluble fraction, insoluble fraction, and protein solubility changes between diseases, followed by enrichment and correlation analysis.

**RESULTS:**

CBD and PiD showed the greatest proteomic similarity in both the soluble and insoluble fractions, while PSP was the most divergent in comparison to other diseases. We observed critical changes in the solubility of lysosomal regulators, postsynaptic proteins, the extracellular matrix (ECM), and mitochondrial proteins.

**DISCUSSION:**

We have contrasted the solubility patterns of proteins across three tauopathies for the first time. Protein solubility differences reveal divergence in disease processes.

**Highlights:**

- Tau isoforms are differentially soluble in primary tauopathies
- PSP proteomics profile was the most divergent of the tauopathies examined
- SORT1 is highly insoluble in CBD and aggregates to different extents in tauopathies
- There are shifts in solubility for key signalling pathways; ROCK1 and JAK2
- Unique lysosomal proteins are more insoluble in distinct tauopathies

## 1 BACKGROUND

Tauopathies encompass a class of neurodegenerative diseases characterized by the abnormal accumulation and aggregation of tau, such as corticobasal degeneration (CBD), Pick's disease (PiD), and progressive supranuclear palsy (PSP).^1^, ^2^, ^3^, ^4^, ^5^ These diseases are predominantly sporadic and constitute a significant proportion of frontotemporal dementia with tau (FTD).^6^ Clinical diagnosis associated with primary tauopathy subtypes is difficult as they have many overlapping clinical symptoms, and few biomarkers exist to differentiate primary tauopathy subtypes from other types of FTD.^7^, ^8^ Autopsy is required to accurately diagnose individual primary tauopathy subtypes. Furthermore, there is no cure for these diseases, although several clinical trials are in progress.^9^

The structure, biochemical properties, and cell type specificity of tau aggregates in each tauopathy are unique.^10^, ^11^, ^12^, ^13^, ^14^ For example, tau can be expressed as protein isoforms containing three (3R) or four (4R) microtubule binding domains.^15^ CBD and PSP tau aggregates are primarily composed of 4R tau, while aggregates in PiD are composed of 3R tau.^16^, ^17^ Each of these diseases preferentially impacts different regions of the brain: CBD most strongly impacts the premotor cortex, parietal lobes, and striatum, often in an asymmetric manner.^18^, ^19^, ^20^ PSP primarily affects the motor cortex and subcortical regions (including globus pallidus, subthalamic nucleus, and striatum).^18^, ^21^, ^22^ In contrast, PiD leads to rapid degeneration of the frontal and temporal lobes.^23^, ^24^ Tau aggregates in CBD are prevalent in neurons and astrocytes (e.g., astrocytic plaques), with some presence of oligodendroglial coiled bodies.^14^, ^25^, ^26^, ^27^ Tau aggregates in PSP also occur in neurons (e.g., globose tangles and pretangles), astrocytes (e.g., PSP‐characteristic tufted astrocytes), and oligodendroglia (coiled bodies).^10^, ^14^, ^27^, ^28^ PiD tau aggregates include pick bodies and ramified astrocytes.^14^, ^28^ Despite these differences, tauopathies share genetic risk factors, including the MAPT haplotype and candidate risk genes MAPT and MOBP.^1^, ^21^, ^29^, ^30^, ^31^ Curiously, the H2 haplotype appears protective for 4R tauopathies (CBD and PSP) but a risk factor for 3R tauopathy (PiD).^1^ These differences in the profiles of tauopathy highlight divergence in the molecular underpinnings of disease that are not well understood.

A significant knowledge gap for the tauopathy field is whether the same disease mechanisms drive pathology in all primary tauopathies or if unique disease mechanisms are at play. In our previous studies, we have found unbiased proteomics of human brain tissue to be an excellent way to comprehensively profile disease mechanisms.^32^, ^33^, ^34^, ^35^ There are surprisingly few prior proteomic studies of primary tauopathy human brain tissue,^36^, ^37^, ^38^, ^39^, ^40^ and no prior studies have compared proteomic differences between 3R and 4R tauopathies. Given that the accumulation of insoluble aggregates is a unifying feature of all tauopathies, we were particularly interested in profiling proteins with changing solubility in tauopathies, as this could provide valuable insight into protein dysfunction and the presence of potential novel co‐pathologies. Therefore, the aim of this study was to profile the sarkosyl soluble and insoluble proteomes in CBD, PiD, and PSP. Sarksoyl is a mild detergent that is capable of solubilizing many natively folded proteins in a cell.^41^ Shifts in solubility can be indicative of biochemical modifications to a protein that render it insoluble or indicate that the protein is present in a detergent‐insoluble aggregate. This approach has been used to assess tau post‐translational modifications that alter its solubility and aggregation propensity in tauopathies.^16^ It has also been used to assess protein solubility changes associated with Alzheimer's disease (AD) pathologies.^42^, ^43^, ^44^ By studying the solubility across CBD, PiD, and PSP, we aim to assess which proteins are uniquely contributing to disease processes and potentially entering an aggregated state. These protein solubility shifts offer insights into disease‐specific processes and potentially generate biomarkers to help differentiate these tauopathies.

**RESEARCH IN CONTEXT**

1. **Systematic review**: The authors reviewed the literature using traditional (e.g., PubMed) sources focusing on prior proteomic studies of primary tauopathies and the roles of proteins of interest. Little is known about comparative changes in protein solubility across tauopathies.
2. **Interpretation**: Our findings highlight the disease specific changes in protein solubility of critical lysosomal and signalling associated proteins. We found the most divergence in PSP patterns of solubility.
3. **Future directions**: This study compares the solubility changes of proteins across primary tauopathies for the first time and provides valuable insight into disease processes. Future studies can build on this new knowledge to determine 1) the mechanistic implications of solubility changes of these proteins in tauopathies, 2) if these protein differences have biomarker potential 3) if insoluble proteins can be chemically shifted back to soluble states as a potential treatment avenue for tauopathies.

## 2 METHODS

### 2.1 Human brain tissue

All procedures were performed under protocols approved by the Sydney University Human Ethics Committee. In all cases, written informed consent for research was obtained from the patient or legal guardian, and the material used had appropriate ethical approval for use in this project. All patients’ data and samples were coded to protect patients’ identities. All tissue used was acquired from the Sydney Brain Bank, Australia. For sarkosyl fractionation, mass spectrometry and Western blot, fresh frozen tissue taken from superior frontal cortex at the level of the head of the caudate nucleus from *n* = 5 patients with CBD, *n* = 5 patients with PiD, and *n* = 5 patients with PSP were used. Our primary inclusion criterion was the presence of tauopathy‐specific pathology, which was determined by histological assessment performed by neuropathologists at the Sydney Brain Bank. Cases with no or minimal co‐pathology were prioritized to the best of our ability (e.g., no or minimal AD neuropathologic change, TDP‐43 pathology, Lewy bodies, aging‐related tau astrogliopathy (ARTAG), primary age‐related tauopathy (PART), hippocampal sclerosis, and small vessel disease). Cases were selected based on neuropathology alone: clinical diagnosis was not considered in case selection but is provided in Table S1 for interest. For Western blot experiments an additional five cases of AD, mild cognitive impairment (MCI) and controls (CTRL) were used as a reference for tau epitopes. For immunofluorescent staining, formalin‐fixed paraffin‐embedded (FFPE) sections were obtained from the superior frontal cortex of the alternate hemisphere of the same cases used for proteomics. Case‐specific details are provided in Table S1.

### 2.2 Tissue homogenization

Tissue homogenization was performed as per previously published methods.^35^ Briefly, 250 mg of grey matter was dissected from frozen tissue blocks of each sample. Tissue was then pulverized on dry ice with a hammer. Pulverized tissue was homogenized in low salt homogenization buffer (50 mM HEPES pH 7.0, 250 mM sucrose, 1 mM ethylenediaminetetraacetic acid [EDTA]), protease inhibitor cocktail (cOmplete mini tablets, EDTA‐free Millipore Sigma), and phosphatase inhibitor cocktail (PhosSTOP EASYpack, Roche) with a Dounce homogenizer. Protein concentration was then determined using a Micro bicinchoninic acid (BCA) assay kit (Thermo Micro BCA Assay Cat. No.: 23235). Homogenized samples were flash‐frozen in a dry ice:ethanol slurry and stored at −80°C.

### 2.3 Western blotting

Western blots were used to profile total brain homogenates of all cases for tau species abundance. A total of 15 µg of total protein was mixed with Bolt LDS sample buffer, 100 mM dithiothreitol (DTT), and boiled for 5 min. Samples were then run on 4%–12% NuPage Bis‐Tris 26‐well midi gels (Invitrogen). Proteins were transferred to 0.2 µm polyvinylidene fluoride (PVDF) and blocked with 5% skim milk or 5% bovine serum albumin (BSA) in Tris‐buffered saline with Tween‐20 (TBS‐T). Blots were probed overnight at 4°C with total tau (Tau‐5 Thermo, MA5‐12808 1:1000), pT217 tau (Thermo, #44‐744, 1:1000), and pTau S199/202 (Thermo, #44‐768G, 1:500). Blots were then incubated for 2 h at room temperature with secondary antibody (anti‐rabbit or anti‐mouse horseradish peroxidase (HRP) conjugated antibody, 1:10,000). Western blots were then developed with electrochemiluminescence (ECL) Western blotting substrate (Merck #WBULS0500) and imaged on a Thermo Scientific CL1500 gel imaging system.

### 2.4 Sarkosyl fractionation

Total homogenates were combined with 10× sarkosyl buffer [5 M NaCl, 10% w/v sarkosyl] to a final concentration of 0.5 M NaCl and 1% sarkosyl. Samples were incubated on ice for 15 min. Samples were then sonicated on ice with a Branson Digital Sonifier SFX150 probe sonicator for 3 × 5‐s pulses using the microtip probe. Protein concentrations were re‐assessed via bicinchoninic acid assay (Pierce). Homogenates were then diluted with 1x sarkosyl buffer to a final concentration of 6 mg/mL. 5 mg (833 µL) of protein was then loaded into 1.5 mL polycarbonate tubes and ultracentrifuged at 4°C for 30 min at 180,000 × *g* (40,100 rpm) with a Sorvall Discovery 90 ultracentrifuge in an F50L‐24 × 1.5 rotor. The supernatant (soluble proteins) was transferred to a new 1.5 mL Eppendorf protein low‐bind tube and flash‐frozen. Pellets were washed with 800 µL of 1× sarkosyl buffer and ultracentrifuged again at 4°C for 30 min at 180,000 × *g*. The supernatant was discarded, and the pellets (insoluble proteins) were flash‐frozen. Samples were stored at −80°C before mass spectrometry.

### 2.5 Protein solubilization and digestion

Each sample was supplemented with lysis buffer to a final concentration of 5% sodium dodecyl sulfate (SDS), 10 mM Tris(2‐carboxyethyl)phosphine hydrochloride (TCEP), 20 mM chloroacetamide (CAA), and 100 mM Tris (pH = 8). Then all samples were incubated in the thermoshaker for 30 min at 90°C (2000 rpm) and centrifuged at 16,500 × *g* for 5 min at room temperature (RT). Supernatants were transferred into 96‐well plates for subsequent processing. Proteins were precipitated on magnetic SP3 beads (250 µg/sample) by 2× dilution with EtOH and then washed on beads three times with 150 µL of 85% EtOH. Enzymatic digestion was performed in 100 µL of 50 mM Tris buffer at pH = 7.4 with 0.2 µg of trypsin (overnight at 37°C and 1000 rpm). The resulting peptides were transferred into clean tubes, the SP3 beads were washed with 100 µL of 0.1% FA, and the eluate was combined with the corresponding peptides. Peptides were loaded onto an Evosep One C18 tip, equilibrated according to the manufacturer's instructions, for subsequent analysis by liquid chromatography‐tandem mass spectrometry (LC‐MS/MS).

### 2.6 LC‐MS/MS

LC separation was performed online on an EVOSEP ONE LC^45^ utilizing a Dr Maisch C18 AQ, 1.9 µm beads (150 µm ID, 15 cm long, cat# EV‐1106) analytical column. Peptides were gradient eluted from the column directly to an Orbitrap HFX mass spectrometer using 88 min extended EVOSEP method (SPD15) at a flow rate of 220 nL/min. The mass spectrometer was operated in data‐independent acquisition mode (DIA)^46^ doing MS^2^ fragmentation across 22 m/z windows after every MS scan event.^45^ High‐resolution full MS spectra were acquired with a resolution of 120,000, an AGC target of 3e6, a maximum ion injection time of 60 ms, and a scan range of 350 to 1650 m/z. Following each full MS scan 22 data‐independent higher‐energy collisional dissociation tandem mass spectrometry (HCD MS/MS) scans were acquired at the resolution of 30,000, AGC target of 3e6, stepped NCE of 22.5, 25 and 27.5. The mass spectrometric raw files are accessible at the MassIVE Repository under accession MassIVE MSV000096544.

### 2.7 Mass spectrometry data analysis

MS data were analyzed using Spectronaut software and searched in directDIA mode against the *H. sapiens* UniProt database. The database search was performed in the integrated search engine Pulsar. For the search, the enzyme specificity was set to trypsin, with the maximum number of missed cleavages set to 2. Oxidation of methionine was searched as a variable modification; carbamidomethylation of cysteines was searched as a fixed modification. The false discovery rate (FDR) for peptide, protein, and site identification was set to 1%. Protein quantification was performed on the MS2^45^ level using the three most intense fragment ions per precursor. Raw label‐free quantitation is provided in Table S2.

### 2.8 Bioinformatics and statistics

Data filtering, imputation, and statistical analysis were performed using R Studio (R v4.4.1) with the tidyverse 2.0.0 package used for general data handling and plotting alongside ggpubr v0.6.0. Principal component analysis (PCA) was performed on raw label‐free quantities (LFQs) to determine sample distributions, after dropping the bottom 30% of variable proteins (i.e., proteins with very little variation as they do not contribute meaningfully to the principal components), using the PCAtools v2.16.0. LFQs were normalized using a modified *z*‐score method. This approach normalizes protein intensities to the median and median absolute deviation, which are both more robust measures of central tendency and sample dispersion.

$$mod\;z-score=\frac{0.6745\left(Protein\;Intensity-Sample\;Median\right)}{MAD}$$

Normalized values were then filtered to be present in at least half of all measurements (81 proteins excluded). Missing values were then imputed using the kNN method (impute v1.78.0).

To determine if covariates, including *post mortem* delay (PMD) and sex, impacted protein quantities, we first assessed if group means were different with one‐way analysis of variance (ANOVA). Means from PMD and age of each group were not different. Next, we correlated all known covariates with each principal component with the “eigencorplot” function in PCAtools on normalized and imputed data. PMD did not correlate with any of the top 10 principal components, suggesting it does not meaningfully alter the variation within this dataset, so we did not control for PMD (Figure S1). Furthermore, PMD did not correlate with the expression of differentially soluble proteins (Figure S1).

Statistical comparisons were performed on the filtered, normalized, and imputed values (Table S3) using linear models generated in limma v3.60.4 and fit with empirical Bayes statistics for differential expression. Contrasts were set up to compare soluble and insoluble protein changes between diseases (e.g., CBD insoluble—PSP insoluble) as well as complex comparisons to assess changes in relative solubility between diseases (e.g., [CBD insoluble—CBD soluble]—[PSP insoluble – PSP soluble]). Multiple comparison correction was performed with the Benjamini–Hochberg method. Significantly altered proteins were defined as those with an absolute log_2_ fold change (FC) > 0.585 (FC > 1.5 or < 0.66) and FDR < 0.05. Volcano plots of all contrasts were generated with EnhancedVolcano v1.22.0, with the x‐axis displaying log_2_ FC and the y‐axis displaying ‐log_10_ PValue. Thresholds were set to absolute log_2_ FC > 0.585 (fold change > 1.5 or < 0.66), and y‐axis thresholds were set at FDR < 0.05.

For the tau peptide level, solubility changes, the total peptide abundances were summed for all mass detections of the same peptide sequence (Table S4). No imputation or normalization was performed on these data. Summed total peptide abundances were plotted for soluble and insoluble fractions in each disease. All protein and peptide level abundances are presented as boxplots where the central marker represents the median, upper and lower hinges represent the 75th and 25th percentiles, respectively, and whiskers represent ± 1.5× the interquartile range. Individual peptide quantities were tested for normality with the Shapiro–Wilks test, then significance was tested with either the Kruskal–Wallis test followed by Dunn's post‐hoc test or one‐way ANOVA followed by Tukey's post‐hoc test.

Protein abundances were correlated with pathology‐related proteins (APP, tau, and α‐synuclein) using bicor rho calculated with the bicorAndPvalue function from the WGCNA package v1.73 individually for each disease subset, but using soluble and insoluble abundances together. Gene set enrichment analysis (GSEA) was performed on all major comparisons. GSEA was limited to gene ontology cellular compartment terms and was performed using the packages, clusterProfiler v4.12.6, enrichplot v1.24.0, ggridges v0.5.6 against the human annotation Org.Hs.eg.db v3.19.1. Gene set enrichments were corrected for multiple comparisons using the Benjamini–Hochberg method. For plotting of GSEA results, enriched sets were simplified with the “simplify” function in clusterProfiler to remove term descriptions with more than 70% similarity. For rank‐shift plots, proteins were ranked based on their average expression and plotted rank versus expression per group (disease and fraction) with selected proteins highlighted to illustrate relative shifts in rank.

Proteins sparsely identified by LC‐MS (detected in < 50% of cases) could not be imputed with the knn method and were filtered out before normalization and imputation. These proteins alongside proteins missing substantially (missing in 80% of samples) from one fraction/group or the other were manually assessed (Table S2). Proteins were called missing when detected in one or fewer samples of any one group (e.g., CBD soluble or CBD insoluble). These data are included to determine if there were any proteins uniquely identifying any one disease or fraction and highlight proteins that may be inappropriately imputed.

All figure panels were created in Adobe Illustrator v28.1.

### 2.9 Fluorescent staining and imaging of human FFPE tissue

FFPE tissue sections from the same cases as used in mass‐spectrometry analysis underwent indirect immunofluorescent staining and imaging using the method described in.^47^ Briefly, sections were deparaffinized and rehydrated through a series of xylene and ethanol washes. Antigen retrieval was achieved by boiling in citrate buffer for 21 min (10 mM sodium citrate, 0.05% Tween‐20, pH 6). Sections were then incubated in proteinase K for 5 min at room temperature (Sigma‐Aldrich, P5568, 0.125 mg/mL). Sections were blocked in 10% normal horse serum and incubated with anti‐Sortilin (Abcam, ab16640, 1:200), anti pTau (Thermo, 44‐768G, 1:500), and anti‐glial fibrillary acidic protein (GFAP) in 4% normal horse serum overnight at 4°C. CF488‐ (Sigma, SAB4600036, 1:1000), AlexaFluor647‐ (Invitrogen, A32787, 1:1000), AlexaFluor750‐ (Jackson, 703‐655‐155, 1:1000) conjugated secondary antibodies and Hoechst 33342 (Sigma, B2261, 1:500) were applied for 2 h at room temperature prior to coverslipping with Antifade ProLong Glass (Invitrogen, P36984). One additional slide was treated without primary antibodies to act as a negative control. Whole slide images were acquired using an Olympus VS200 Slide Scanner at 20× magnification (NA 0.8). Representative 60x (NA 1.4) images were captured on a Nikon C2 Confocal microscope. Low‐power images primarily spanning layers II‐V of the superior frontal cortex were extracted with QuPath (v0.4.4). Images were exported as “.tif” images. Images were processed in ImageJ (v2.14.0), and the empty channel λ568 was subtracted from all other channels to minimize autofluorescent signal. Images were constructed in Adobe Photoshop (v26.4.1), and uniform thresholds were applied to each channel of all images; the final figures were made in Adobe Illustrator (v28.1).

## 3 RESULTS

### 3.1 Proteomics overview

**FIGURE 1:**
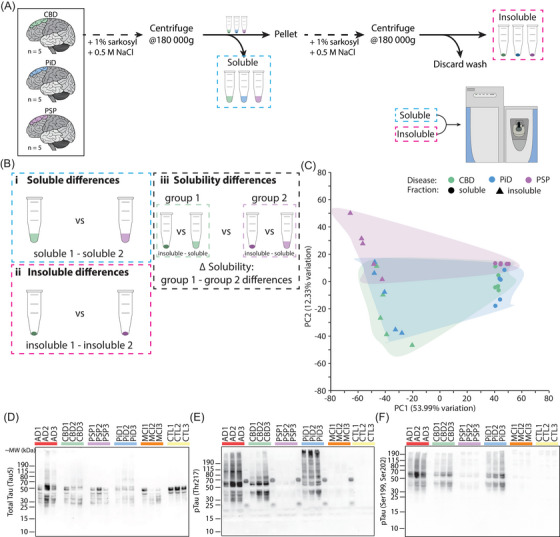
Proteomics approach and PCA of tauopathy soluble and insoluble proteins. (A) Overview of the methods used to isolate soluble and insoluble proteins for mass‐spectrometry. (B) Example statistical contrasts between CBD and PSP. Overview of the statistical comparisons made comparing differences in (1) soluble abundances between tauopathies, (2) insoluble abundances between tauopathies, and (3) differences in solubility between tauopathies. (C) PCA plot of raw proteomics data. Demonstrates a clear division between soluble and insoluble data, with disease separated on PC2. (D–F) Western blots showing the relative amount of total tau (Tau‐5), pT217 tau, and pS199/202 tau. Three of five cases are shown. Western blots of the remaining two cases can be seen in Figure S2. CBD, corticobasal degeneration; PCA, principal component analysis; PSP, progressive supranuclear palsy; PiD, Pick's disease; AD, Alzheimer's disease; MCI, mild cognitive impairment; CTL, controls

Our primary aim was to identify the protein differences in the soluble and insoluble fractions from three primary tauopathies: PSP, CBD and PiD. Soluble and insoluble proteins were isolated using sarkosyl fractionation and analyzed using mass spectrometry‐based proteomics (Figure 1A). We quantified 5478 proteins across the soluble and insoluble fractions for each disease (Table S2). Western blot was used to initially profile the abundance of total tau, tau phosphorylated at threonine 217 (pT217) and tau phosphorylated at serine 199/202 (pS199/202) in the total brain homogenate from each case. Additional cases of advanced AD, MCI and age‐matched cognitively normal controls (CTRL) were included to provide additional context. Total tau levels were largely similar across the tauopathies. CBD and PiD had similar abundance for both phosphorylated tau (pTau) species tested, albeit with different distributions across the molecular weight gradient (Figure 1C, Figure S2). In contrast, PSP had comparatively little pTau, with both epitopes (pT217 and pS199/S202) appearing similar to controls (Figure 1C, Figure S2). This finding is consistent with our previous publication that showed PSP had the least AT8 pTau levels in the frontal cortex of the same cases using immunofluorescence.^48^

Mass spectrometry was then used to assess the proteome of soluble and insoluble fractions isolated from all PSP, CBD, and PiD cases. PCA showed a clear separation of soluble and insoluble fractions (Figure 1C), validating that our approach successfully isolated distinct pools of proteins in each fraction. A comparison of protein expression between the three tauopathies showed that PSP had the most unique protein expression, while CBD and PiD were more similar (Figure 1C). This was the case for both soluble and insoluble fractions.

### 3.2 Tau peptide solubility patterns reveal isoform‐specific insolubility

**FIGURE 2:**
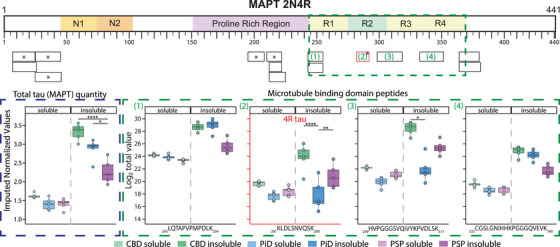
MAPT peptide level solubility changes highlight bias to 4R tau in CBD insoluble fraction. Differential solubility of peptides mapped to MAPT 2N4R. The MTBR is highlighted (green dashed line). Black boxes represent each of the 15 peptides detected. Blue dashed box shows MAPT protein level normalized abundances across diseases. Green dashed box shows detected levels of each MAPT peptide from the microtubule binding domain. MTBR peptide 3 is highlighted red as it is the unique peptide for 4R tau (excluded from 3R tau). * in black boxes represent peptides that had significant differences in one fraction between diseases. Significance is denoted by *’s in boxplots, * FDR < 0.05, ** FDR < 0.01, *** FDR < 0.001, **** FDR ≈ 0. CBD, corticobasal degeneration; FDR, false discovery rate; MTBR, microtubule binding domain

To determine if there were biases in the insolubility of tau isoforms across the tauopathies, we assessed the solubility of each tau peptide detected (Table S4). We detected 15 peptides mapping to 2N4R tau (Figure 2). One peptide was detected on the boundary of the microtubule‐binding domain repeat 2. This domain gets alternatively spliced to produce 3R (domain excluded) or 4R tau (domain included) tau. Thus, the inclusion of peptide _281_KLDLSNVQSK_290_ defines the presence of 4R tau. The _281_KLDLSNVQSK_290_ peptide was significantly more abundant in the insoluble fraction of CBD than PiD (ANOVA adjusted *p‐*value = 0.00005) but not significantly more than PSP (ANOVA adjusted *p‐*value = 0.068, Figure 2). Similarly, PiD and PSP were not significantly different (ANOVA, adjusted *p‐*value = 0.062). In contrast, soluble _281_KLDLSNVQSK_290_ was similar between each disease. These results confirm the preferential accumulation of insoluble 4R tau in CBD, but not in PiD. PSP had a lower degree of insoluble 4R tau, aligning with the low levels of aggregated tau observed via Western blot (Figure 1C). Only _195_SGYSSPGSPGTPGSR_207_ had significantly different abundances between PiD and PSP (ANOVA, adjusted *p‐*value = 0.016).

### 3.3 Soluble proteome comparison

**FIGURE 3:**
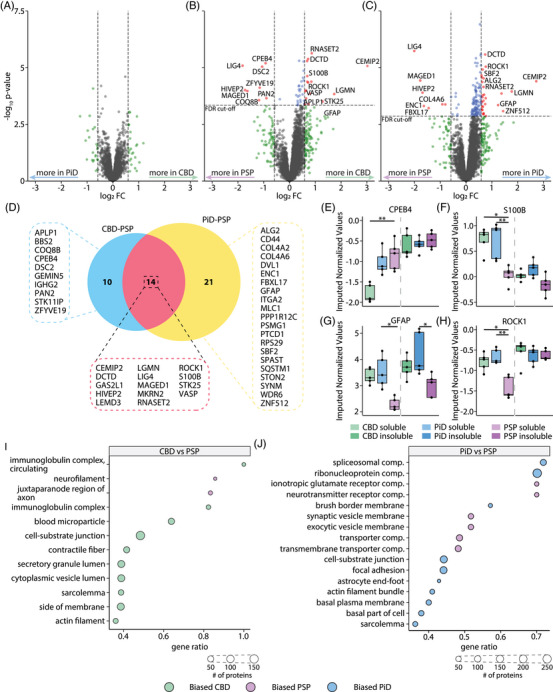
Changes in soluble protein abundance between tauopathies highlight differences in the cytoskeleton, cell junctions, and spliceosome between diseases. Volcano plots showing proteins with increased abundance in the soluble fraction in comparisons between (A) CBD‐PiD, (B) CBD‐PSP, and (C) PiD‐PSP. (D) Venn diagram showing the overlap of differentially abundant soluble proteins in each comparison boxplot plots of normalized protein expression for example proteins, (E) CPEB4, (F) S100B, (G) GFAP, and (H) ROCK1. (I) GSEA enrichments for proteins enriched more in CBD or PSP soluble comparisons (J) GSEA enrichments for proteins enriched more in PiD or PSP soluble comparisons. GSEA results for CBD‐PiD are in Table S8. Significance is denoted by *’s in boxplots, * FDR < 0.05, ** FDR < 0.01, *** FDR < 0.001, **** FDR ≈ 0. CBD, corticobasal degeneration; GSEA, gene set enrichment analysis; PiD, Pick's disease; PSP, progressive supranuclear palsy

Comparison of the abundance of proteins in the soluble fraction between diseases identified no protein differences in CBD versus PiD, 24 protein differences in CBD versus PSP and 35 protein differences in PiD versus PSP (threshold FDR < 5% and |log_2_FC| > 0.584; Figure 3A–C; Table S5–S8). The lack of individual protein differences between CBD and PiD was consistent with PCA results, suggesting that the soluble proteome of the two diseases were similar. Fourteen proteins showed similar soluble abundance differences when comparing CBD to PSP and PiD to PSP (Figure 3D). Interesting examples of soluble abundance changes in specific diseases included CPEB4, S100B, GFAP, and ROCK1. CPEB4 was significantly reduced in CBD in comparison to both PSP and PiD and is important for ribonucleoprotein complex formation and RNA translation (Figure 3E).^49^ S100B was lower in PSP in comparison to both CBD and PiD (Figure 3F), suggesting heightened CNS distress in CBD and PiD.^50^ GFAP was significantly more abundant in both CBD and PiD compared to PSP (Figure 3G) suggesting differential impacts on astrocytes between PSP and CBD/PiD. Similarly, ROCK1 was significantly more abundant in the soluble fraction of both CBD and PiD compared to PSP (Figure 3H), suggesting impacts on the JAK2‐STAT3 pathways or altered autophagy and apoptosis pathways through differential phosphorylation of Beclin‐1/2.^51^, ^52^, ^53^

To determine which pathways may be impacted by the differential abundance of proteins in the soluble fraction, we performed GSEA. In the CBD versus PSP comparison, CBD is biased toward pathways involved in cytoskeletal and ECM maintenance. Furthermore, we saw enrichments for vesicle lumen and secretory granules, suggesting changes in exocytic components in CBD compared to PSP. PSP enriched only two terms in comparison to CBD soluble protein abundances, neurofilament, and the juxtaparanode region of the axon, suggesting that neurons are remodeling axonal segments.

In PiD versus PSP, pathways biased to PiD included spliceosome compartments and cell–cell junctions (including the astrocyte end‐foot, which encapsulates all blood vessels, Figure 3J, Table S8). PSP‐biased pathways included synaptic processes, including neurotransmitter complexes (glutamate) and synaptic/exocytic vesicle cycles (Figure 3J, Table S8). This suggests that PSP has alterations occurring in axonal segments when compared to CBD and alterations in protein activity at the post‐synapse when compared to PiD in the frontal cortex.

### 3.4 Insoluble proteome comparison

**FIGURE 4:**
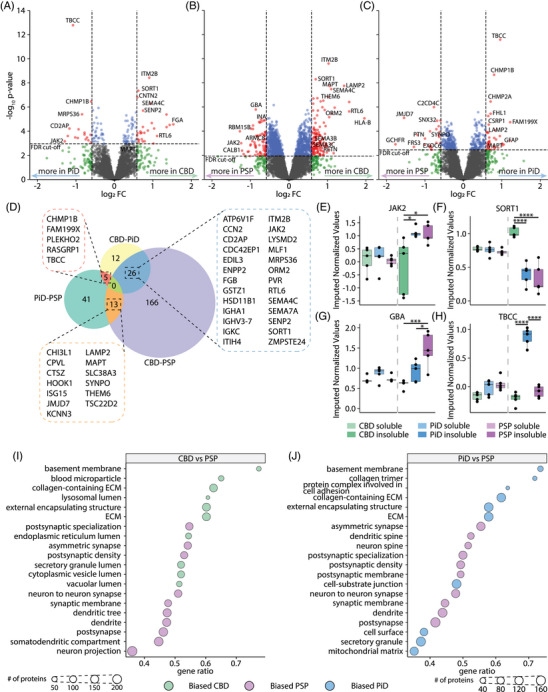
Changes in insoluble protein abundance between tauopathies highlight differences in the lysosomal processes, post‐synapse, and ECM proteins between diseases. Volcano plots showing proteins with increased abundance in the insoluble fraction in comparisons between (A) CBD‐PiD, (B) CBD‐PSP, and (C) PiD‐PSP. (D) Venn diagram showing the overlap of differentially abundant soluble proteins in each comparison boxplot of normalized protein expression for example proteins, (E) JAK2, (F) SORT1, (G) GBA, and (H) MAPT. (I) GSEA enrichments for proteins enriched more in CBD or PSP soluble comparisons (J) GSEA enrichments for proteins enriched more in PiD or PSP soluble comparisons. GSEA results for CBD‐PiD are in Table S12. Significance is denoted by *’s in boxplots, * FDR < 0.05, ** FDR < 0.01, *** FDR < 0.001, **** FDR ≈ 0. CBD, corticobasal degeneration; ECM, extracellular matrix; FDR, false discovery rate; GSEA, gene set enrichment analysis; PiD, Pick's disease; PSP, progressive supranuclear palsy

Comparisons of the insoluble fraction protein abundances highlighted more extensive disease‐associated differences. Contrasts between the insoluble fractions identified 43 proteins in CBD‐PiD, 205 proteins in CBD‐PSP and 59 proteins in PiD‐PSP with differential insoluble abundance (threshold FDR < 0.05, |log_2_FC| > 0.584, Figure 4A–C, Tables S9–S12). CBD versus PSP showed the greatest difference in insoluble proteomes. Key examples of proteins enriched in the insoluble fractions included JAK2, SORT1, GBA, and tau. JAK2 was more insoluble in PiD and PSP compared to CBD (Figure 4E). SORT1 had drastically increased relative abundance in CBD compared to either PiD or PSP (Figure 4F). Increased SORT1 insolubility could suggest increased membrane portions leading to decreased progranulin levels and altered lysosomal activity and cycling. Interestingly, the Parkinson's disease risk gene GBA was more abundant in PSP insoluble fraction and is significantly more abundant than in CBD (log_2_FC = −0.86, FDR = 0.0005), and was significant but did not pass magnitude thresholds when compared to PiD (log_2_FC = −0.53, FDR = 0.028). Importantly, tau was less abundant in the insoluble fraction of PSP compared to CBD and PiD (Figure 4H). This confirms both the Western blots (Figure 1C) and our previously published data that there is less insoluble tau in the PSP frontal cortex compared to other tauopathies.^48^

GSEA revealed a strong bias toward extracellular matrix (ECM) components and vesicle lumen proteins being more abundant in the insoluble fraction of CBD than PSP (Figure 4I). In contrast, proteins with increased abundance in the insoluble fraction of PSP were biased much more strongly toward the postsynaptic and dendritic compartments (Figure 4I, Table S12). Similarly, the lists of protein insolubility abundance changes in PiD‐PSP highlighted the same enrichment biases, abundant insoluble proteins in PSP favoring postsynaptic/synaptic proteins, and in PiD, these proteins favor the ECM compartment (Figure 4J, Table S12). However, proteins with greater abundance in the PiD insoluble fraction were enriched more strongly for mitochondrial processes than PSP.

### 3.5 Solubility shifts

**FIGURE 5:**
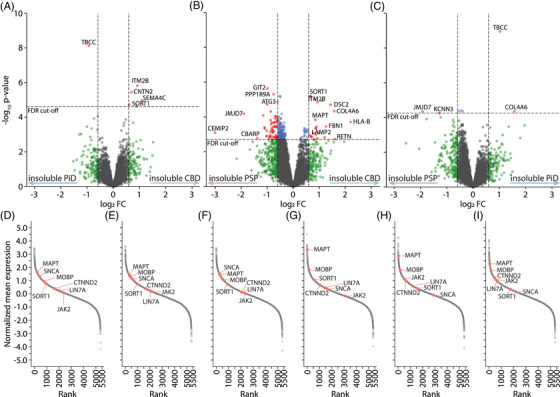
Changes in solubility between diseases detected by complex contrasts. Volcano plots show proteins with changes overall solubility in comparisons between (A) CBD and PiD, (B) CBD and PSP and (C) PiD and PSP. (D–F) rank plots of proteins identified in the soluble fractions of CBD, PiD and PSP, respectively. (G–I) Rank plots of proteins identified in the insoluble fraction of CBD, PiD and PSP, respectively. CBD, corticobasal degeneration; PCA, principal component analysis; PiD, Pick's disease; PSP, progressive supranuclear palsy

Finally, to determine what proteins have genuine changes in solubility between diseases, we assessed the complex statistical contrast of differences between solubility rates in each disease (analysis iii, Figure 1B). This was done because the different sample preparation methods used to isolate soluble and insoluble fractions meant that direct contrasts between soluble and insoluble fractions within a disease group were inappropriate. CBD‐PiD contrasts showed six proteins had solubility differences, five of which were more insoluble in CBD (Figure 5A, Table S13). This included SORT1, CNTN2, ITM2B, SENP2, and SEMA4C which were also comparatively more insoluble in CBD than PSP. The CBD‐PSP contrast identified 78 proteins that showed differing shifts in solubility between diseases (Figure 5B, Table S14). This included multiple proteins important for autophagy and lysosomal activity (GBA1, ATG3, ARL8B) that were more insoluble in PSP in comparison to CBD. Proteins involved in synapse formation and function also were more insoluble in PSP, including the synaptic regulator CTNND2, CBARP7 which regulates calcium channels, and LIN7A which regulates axonal development and distribution of channels in the synapse.^54^ PiD only showed preferential insolubility of 2 proteins (TBCC and COL4A6, Tables S13, S15). This may be explained by the fact that PiD expression patterns typically lay midway between CBD and PSP. These solubility changes are demonstrated in rank‐shift plots (Figure 5D–I). MAPT moves from ∼ rank 227, 334 and 311 in soluble fractions to rank 9, 20, and 62 in the insoluble fractions of CBD, PiD, and PSP, respectively (Figure 5D–I). JAK2 becomes significantly more soluble in CBD, shifting from rank 2380 in soluble fractions to 3088 in insoluble fractions. In contrast, JAK2 moves from rank 2166 and 2340 in soluble fractions to rank 503 and 577 in insoluble fractions of PiD and PSP, respectively. SORT1 shows the opposite pattern, with rank increasing dramatically from soluble to insoluble fractions in CBD (Figure 5D,G). GSEA highlighted the ECM and fibrinogen complexes becoming more insoluble in CBD compared to PSP (Figure S3, Table S16). While in PSP, proteins involved in neuronal projections and postsynaptic densities were becoming preferentially insoluble compared to CBD (Figure S3, Table S16).

### 3.6 Common disease protein pathologies are not different between tauopathies

**FIGURE 6:**
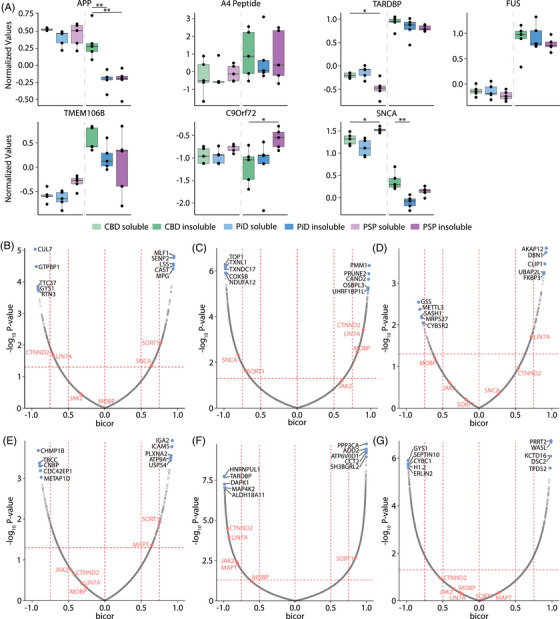
Alternative disease pathologies are not differentiable across tauopathies. MAPT and SNCA correlations vary across tauopathies. (A) Disease‐associated pathological proteins are not significantly altered across tauopathies. (B–D) Bicor analysis shows different patterns for correlation with MAPT in CBD, PiD, and PSP, respectively. (E–G) Bicor plots assessing protein correlations with SNCA in CBD, PiD, and PSP, respectively. The top five positively and negatively correlated genes are labelled. MAPT and SNCA are removed from their respective bicor plots as they have a correlation of 1 with themselves. Significance is denoted by *’s in boxplots, * FDR < 0.05, ** FDR < 0.01. CBD, corticobasal degeneration; FDR, false discovery rate; PCA, principal component analysis; PiD, Pick's disease; PSP, progressive supranuclear palsy; SNCA, α‐synuclein

To determine if any other protein pathologies could be driving changes in these cases, we assessed the solubility shifts of other commonly aggregating disease‐associated proteins. This list included APP, Aβ (measured using the LVFFAEDVGSNK peptide that corresponds to amino acids 17‐28 of Aβ), TDP43 (TARDBP), FUS, TMEM106B, C9Orf72, and α‐synuclein (SCNA) (Figure 6A). TDP43, FUS, and TMEM106B were typically more abundant in the insoluble fraction, while α‐synuclein was more abundant in the soluble fraction (Figure 6A). In the soluble fraction, we observed significant differences in abundance of both α‐synuclein (log_2_ FC = −0.41, FDR = 0.021) and TDP43 (log_2_ FC = 0.35, FDR = 0.045) in comparisons between PiD and PSP, albeit at the low magnitude of change. In the insoluble fraction, we saw significant abundance differences between CBD and PiD for α‐synuclein (log_2_ FC = 0.47, FDR = 0.005), and between CBD and PSP for C9Orf72 (log_2_ FC = −0.61, FDR = 0.027) and APP (log_2_ FC = 0.55, FDR = 0.002). Only C9orf72 had an increase in abundance above our thresholds (|log_2_ FC| > 0.584, FDR < 0.05), but it is worth noting that it has an abundance below the median value in all cases (Figure 6A). However, contrasts comparing the change in solubility (Figure 1B, analysis iii) did not demonstrate changes in overall solubility between diseases for any of these proteins, suggesting that none of these neurodegenerative disease‐associated proteins are becoming more insoluble (likely aggregating) in any one tauopathy in our cohort.

### 3.7 Protein correlations with tau and α‐synuclein across tauopathies

To determine how tau and α‐synuclein correlate with protein abundances in soluble and insoluble fractions we assessed the bicorrelation of all proteins with tau and α‐synuclein. We were interested in determining if α‐synuclein correlated with protein solubility as we observed an increased abundance of α‐synuclein in our pathologically confirmed PSP cases. Correlations of proteins across the soluble and insoluble fractions with disease associated proteins may indicate proteins that are being pulled into the insoluble fraction alongside pathology or have their biochemical state (phosphorylation etc.) disrupted by pathological processes. PiD had the strongest correlations between tau and other proteins across the soluble and insoluble fractions (1618 proteins *ρ* > 0.7 and 1639 < −0.7). CBD and PSP had comparatively few strongly correlated proteins (CBD *ρ*: 236 > 0.7, 210 < −0.7, PSP *ρ*: 105 > 0.7 and 19 < −0.7, Figure 5B–D, Table S17). This may indicate a more robust impact of tau aggregation on protein solubility in PiD than in CBD or PSP. Each disease showed unique correlation patterns for proteins with tau solubility. For example, MOBP, a tauopathy risk gene, was strongly correlated with tau in PiD (*ρ* = 0.82; *p‐*value = 0.004) but not in CBD (*ρ* = 0.0; *p‐*value = 0.94) or PSP (*ρ* = −0.61; *p‐*value = 0.06), suggesting tauopathy risk genes are partitioning in solubility differently in each disease. The opposite patterns were observed with the PSP risk factor gene C4A, where CBD had a significant positive correlation between tau soluble/insoluble and C4A (*ρ* = 0.69, *p‐*value = 0.023) compared to PiD's negative correlation (*ρ* = −0.83, *p‐*value = 0.003) and no significant correlation with levels in PSP (*ρ* = 0.36, *p‐*value = 0.31).

Similar patterns were observed in the correlation of α‐synuclein soluble and insoluble levels across each tauopathy. PiD again had the greatest number of strong correlations (1819 proteins *ρ* > 0.7 and 1831 *ρ* < −0.7). PSP protein abundance in the soluble and insoluble fractions had numerous strong correlations with α‐synuclein abundance (359 proteins *ρ* > 0.7 and 921 proteins *ρ* < −0.7), while CBD had few proteins correlated with α‐synuclein (98 proteins *ρ* > 0.7, 85 *ρ* < −0.7). In contrast to tau correlations, MOBP was not significantly associated with α‐synuclein abundance in any disease (Table S17). Curiously, tau levels were positively correlated with α‐synuclein in CBD (*ρ* = 0.65, *p‐*value = 0.043), but negatively correlated with α‐synuclein levels in PiD (*ρ* = −0.81, *p‐*value = 0.005) and PSP (*ρ* = 0.26, *p‐*value = 0.46). These data suggest that protein abundance changes in soluble and insoluble fractions from PSP with minimal pathology may be more closely associated with a combination of α‐synuclein and tau‐related processes, while CBD is largely associated with tau changes. PiD may be strongly influenced by interactions between both tau and α‐synuclein.

### 3.8 Proteins missing from one or more groups

Our filtering approach and imputation mean some proteins that are sparse in one group may be missed in our statistical analysis. To determine if any such proteins existed, we filtered the raw dataset (Table S2) for proteins that were marked “sparse” or “Shared in > 50% of the runs.” We then filtered these further to proteins with no more than one detection in any group but present in the majority of cases in other groups. PSP had notably more proteins that were not detected in the soluble fraction in comparison to other tauopathies (567 ± 140, Table S18). Interestingly, the number of detected proteins in the insoluble fraction was similar between diseases, with fewer cases showing missing values. Most proteins with “sparse” detections in both the soluble and insoluble fractions were of comparatively low abundance (average log_2_(LFQ) of filtered set = 15.66 vs. 17.84 for all proteins).

**FIGURE 7:**
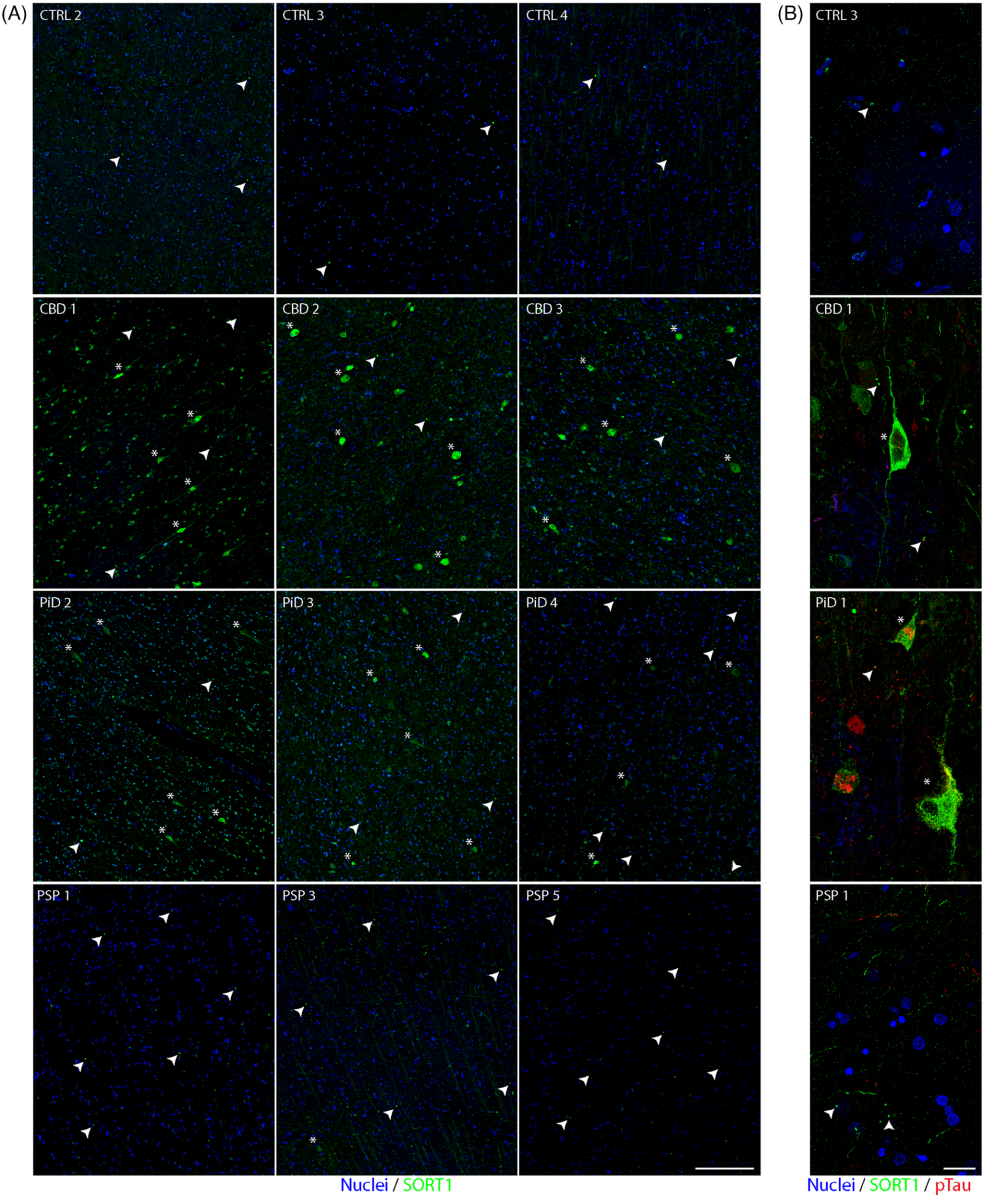
Immunofluorescence staining of human *post mortem* FFPE tissue reveals aggregation of SORT1. (A) Low power images primarily spanning cortical layers II‐V of the superior frontal cortex obtained from three control, CBD, PiD, and PSP cases showing SORT1 (green) and Hoechst nuclear stain (blue). (B) Confocal images (60x, 200 nm pixel size) of control, CBD, PiD, and PSP cases stained for SORT1, showing SORT1 (green), phosphorylated tau (red), and Hoechst nuclear stain (blue). Arrows indicate small SORT1 puncta, and stars indicate neurons with distinct intraneuronal accumulation of SORT1. Scale bar for A is 200 µm. Scale bar for (B) is 20 µm. CBD, corticobasal degeneration; FFPE, formalin‐fixed paraffin‐embedded; PCA, principal component analysis; PiD, Pick's disease; PSP, progressive supranuclear palsy

Key examples of group‐specific proteins included TRIM44, which was only detected in CBD (5/5 cases for both soluble and insoluble fractions) and not detected in PiD or PSP (Figure 7). This may suggest alterations to autophagy specific to CBD.^55^ FZD7 was another example, which was detected in the CBD insoluble fraction, but was missing from the PSP insoluble fraction (Table S18). These represent proteins that show differential insolubility between diseases.

### 3.9 Immunofluorescence of SORT1 reveals disease‐associated changes in distribution and aggregation

We performed immunofluorescent staining and imaging of FFPE *post mortem* brain tissue from CBD, PiD, PSP, and age‐matched control cases (*n* = 5/group) to assess SORT1 expression patterns. Immunostaining revealed diverse SORT1 distribution in tauopathies, ranging from predominantly neuronal to mixed neuronal and astrocytic localization. CBD exhibited the most intense staining, with frequent SORT1‐positive aggregates observed in neurons and astrocytes (Figure 7A, B). SORT1 aggregates in the CBD were particularly dense, showing intense staining (Figure 7B). Two cases of CBD had strong staining of SORT1 in astrocytes in addition to SORT1‐positive aggregates. In contrast, control cases had the lowest overall intensity of SORT1, which was primarily restricted to neuronal and astrocytic processes (Figure 7). PSP and PiD cases displayed similar staining patterns, with aggregate‐like SORT1 puncta observed in neuronal cell bodies, some of which appeared hollow under confocal microscopy (Figure 7), differing from the dense puncta observed in CBD. Additionally, we observed infrequent clusters of neurons with widespread SORT1 expression in PiD and, rarely, in PSP. Nuclear SORT1 staining was occasionally present across all groups.

## 4 DISCUSSION

Here, we report the first comparison of relative protein solubility changes across three tauopathies. We observed multiple disease‐specific changes that may underlie disease processes. Proteins enriched in the sarkosyl insoluble fraction are likely to either have shifted biochemical properties or be present in aggregates. In neurodegenerative diseases, protein insolubility has typically been linked to pathological protein aggregation; however, changes in protein solubility are likely more complex than this. Shifts in solubility are not strictly deleterious; for example, heat‐shock proteins and ECM proteins are expected to become insoluble in their normal function.^56^, ^57^ One example of this is the formation of stress granules, where proteins and mRNA become insoluble during stress. Interestingly, the proteins involved in stress granule formation are stress and cell‐type‐dependent.^58^, ^59^ Importantly, dysregulation of the formation and reversal of these insoluble deposits can trigger neurodegenerative processes.^60^ While the interpretation of solubility changes can be complex, these changes provide insights into the biochemical state and potential aggregation of proteins in disease.

While PSP had the most differences with other tauopathies, a caveat to these results was the lower degree of tau aggregation in that PSP frontal cortex, making it appear more like control cases in Western blots and immunostaining of FFPE tissue. This is expected given the different region‐specific vulnerability for each tauopathy and the reported lower levels of pTau or high‐molecular‐weight tau species in the frontal cortex in PSP.^16^ Notably, our recent study confirmed PSP‐specific tau pathology is still present in frontal cortex of the same cases used in this study, albeit at lower levels than observed in other brain regions (e.g., motor cortex) in PSP or than other tauopathies.^48^ This highlights an important limitation with regards to direct comparison of multiple primary tauopathies, as selection of a single brain region with similar levels of pathology in all three tauopathies is difficult. Here, we focused on a single brain region to limit the complexity of analysis, achieve a similar background of cell types and have some pathology present in all three conditions. This impacted the comparisons of tau species (both phosphorylated and aggregated) but still allows for important findings on disease differences. A further limitation was the exclusion of control tissue to determine which changes are purely disease associated. While comparison to control samples would provide additional context, the between‐disease differences described here still offer meaningful insights.

A peptide level analysis of tau solubility revealed a bias in CBD toward 4R isoforms being preferentially insoluble, despite similar levels of tau isoforms in the soluble fraction of all diseases. Interestingly, the levels of 4R tau (and tau in general) did not differ between PSP (4R‐biased aggregates) and PiD (3R‐biased aggregates). The lack of 4R tau elevation in PSP was unexpected and likely due to the low levels of tau aggregates in our PSP cases in the frontal cortex. This is supported by recent studies comparing the solubility of tau between AD, CBD, PiD, and FTLD that showed 4R tau was insoluble in CBD, but they also detected a difference in soluble 4R tau abundance.^16^, ^61^ Mutation in tau intronic regions alters the splicing balance of 4R tau in FTLD, but we do not see protein‐level evidence of differential isoform expression in our samples.^62^, ^63^ Other studies have shown that 4R tau has a higher aggregation propensity than 3R tau.^64^, ^65^, ^66^ This would indicate that the aggregation of 3R or 4R tau is influenced by either cell‐type‐specific imbalances in 3R/4R abundance or initial aggregation at sites where one tau isoform is prevalent.

In this study, we have detected multiple solubility changes between diseases. We see increases in soluble abundance of proteins that indicate generic glial stress and brain injury, such as S100B in CBD and PiD.^50^, ^67^, ^68^ This is supportive of neurodegenerative impacts in CBD and PiD, while PSP frontal cortex signatures indicate a prestressed state. We do, however, observe changes in neuronal processes in PSP. GSEA reveals greater solubility of neurofilament‐associated proteins (NEFL, NEFM, NEFH, INA, SHANK2, Table S8), postsynaptic and synaptic vesicle‐associated proteins (e.g., SYNGR1, SYNGR3, BSN; Table S8), suggesting there are signs of early neuronal dysfunction. Together, these changes may indicate broad synaptic dysregulation in PSP.

We observed increased levels of GFAP in CBD and PiD which is likely indicative of increased glial reactivity response to widespread pathology. GFAP levels were decreased in both soluble and insoluble fractions in PSP compared to CBD and PiD, most likely due to the limited tau pathology present in the frontal cortex. We see evidence of altered lysosomal activity in tauopathies, with GBA being strongly enriched in the insoluble fraction in PSP while both CBD and PiD have relatively little insoluble GBA. In contrast, LAMP2 is enriched in the insoluble fractions of CBD and PiD. Both GBA and LAMP2 are lysosomal membrane‐associated proteins, so the shifts in opposite directions of solubility are curious. The reduction of insoluble LAMP2 in PSP may indicate normal autophagosome activity (high turnover leading to lower abundance and mostly soluble fraction enriched), while GBA increased abundance in the insoluble fraction may indicate increased lipid turnover activity.^69^

Another key lysosomal‐associated change in solubility was SORT1. SORT1 is a receptor for progranulin and downregulates its activity.^70^, ^71^ This prevents the production of activated granulins, which can degrade protein aggregates involved in neurodegeneration.^72^ We observed differential profiles of SORT1 solubility between CBD and PSP/PiD. In CBD, there is a greater abundance of SORT1 in the insoluble fraction, while PiD and PSP both have substantially less insoluble SORT1. Granulin precursor proteins were only observed in the soluble fraction of all cases, and while not statistically assessed here, appeared to have the highest abundance in CBD soluble fractions (Table S18). These data suggest that SORT1 in CBD may be stably insoluble and may interact more frequently with progranulin. Our SORT1 immunostaining supported our proteomic results, where we observed frequent aggregates of SORT1 in CBD brains. PiD had an intermediate degree of SORT1 aggregation, while PSP had some aggregation compared to controls. These aggregates were predominantly observed in neurons and astrocytes. Intriguingly, SORT1 aggregates have also been reported in AD and PART, where they resemble plaques and intraneuronal granulovacuolar degeneration.^73^, ^74^, ^75^ We observed that SORT1 staining was independent of phosphorylated tau aggregates, suggesting this pathology may be occurring independently of tau pathology. An antibody drug is in development for FTD to increase progranulin production by blocking SORT1.^70^, ^76^ The differential solubility and activity of SORT1 may have implications for which FTD subtypes such a therapy would be effective. The combination of changes in SORT1 and GBA may predispose these regions to greater tau aggregation, but more work is required to untangle this relationship.^72^

We observed solubility changes in signaling pathways mediated by ROCK1 and JAK2. ROCK1 had markedly less protein in the soluble fraction of PSP compared to CBD and PiD, suggesting low activity of ROCK1 in PSP. Insoluble abundance was similar between each disease. ROCK1 can phosphorylate JAK2 to initiate the JAK2/STAT3 signaling cascade^53^, ^77^ or alter autophagy dynamics by phosphorylating beclin 1 and 2.^51^, ^52^ In the case of JAK2, phosphorylation likely increases its solubility and allows for the translocation of JAK2/STAT3 to the nucleus.^77^ In line with ROCK1 soluble/insoluble abundances in PSP, we saw no increase in JAK2 solubility. In PiD, we observed similar patterns of soluble/insoluble abundance of JAK2 as PSP. However, CBD had a lower insoluble abundance of JAK2 and an increase in the soluble fraction, suggesting that JAK2 is more active in CBD and may be activated by ROCK1. We saw no changes in Beclin 1/2, suggesting that the solubility of these proteins was not altered by ROCK1. These signaling pathways are important regulators of energy homeostasis and could have a unique role in CBD.

Studying the shifts in solubility of proteins has highlighted multiple disease‐specific differences. These protein solubility shifts may act as disease‐specific biomarkers; however, further work is needed to determine if these are viable candidates. First, the proteins would need similar shifts in solubility or significant alterations in abundance in another tissue or biofluid (e.g., CSF) that is more accessible than brain tissue. Second, we need more rapid protocols for identifying solubility changes in a clinic that do not require ultra‐centrifuges and lengthy isolation protocols. Recently, SORT1 was reported to be uniquely present in the CSF of FTD patients with C9Orf72 mutations, indicating its potential use as a discriminatory biomarker.^78^

In conclusion, our data reveal novel changes that may be driving disease processes in tauopathies. Interestingly, we found that the overall proteomic profiles between CBD and PiD were very similar, while PSP was the most divergent. Future studies could focus on these differences to better understand tauopathy mechanisms. Our initial immunofluorescence study suggests SORT1 is aggregating in neurons in CBD, but more work is needed to elucidate the impact of these changes. These differences also offer unique opportunities to tailor treatments or biomarkers for individual tauopathies.

## CONFLICT OF INTEREST STATEMENT

The authors declare no conflicts of interest. Author disclosures are available in the Supporting Information.

## CONSENT STATEMENT

This research project was approved by the Human Research Ethics Committee of the University of Sydney and complies with the statement on human experimentation issued by the National Health and Medical Research Council of Australia. Tissues were selected from a neuropathological series collected by the Sydney Brain Bank through regional brain donor programs in Sydney, Australia. The brain donor programs hold approval from the Human Research Ethics Committees of the South Eastern Sydney Area Health Services and comply with the statement on human experimentation issued by the National Health and Medical Research Council of Australia. In all cases, written informed consent for research was obtained from the patient or legal guardian.

## Supporting information



Supporting Information

Supporting Information

Supporting Information

## References

[1] Valentino RR , Scotton WJ , Roemer SF , et al. MAPT H2 haplotype and risk of Pick's disease in the Pick's disease International Consortium: a genetic association study. Lancet Neurol. 2024;23(5):487‐499. doi:10.1016/S1474-4422(24)00083-8 38631765 PMC11877577

[2] Chen JA , Chen Z , Won H , et al. Joint genome‐wide association study of progressive supranuclear palsy identifies novel susceptibility loci and genetic correlation to neurodegenerative diseases. Mol Neurodegener. 2018;13(1):41. doi:10.1186/s13024-018-0270-8 30089514 PMC6083608

[3] Kouri N , Ross OA , Dombroski B , et al. Genome‐wide association study of corticobasal degeneration identifies risk variants shared with progressive supranuclear palsy. Nat Comm. 2015;6(1):7247. doi:10.1038/ncomms8247 PMC4469997 26077951

[4] Yokoyama JS , Karch CM , Fan CC , et al. Shared genetic risk between corticobasal degeneration, progressive supranuclear palsy, and frontotemporal dementia. Acta Neuropathol. 2017;133(5):825‐837. doi:10.1007/s00401-017-1693-y 28271184 PMC5429027

[5] Spillantini MG , Goedert M . Tau pathology and neurodegeneration. Lancet Neurol. 2013;12(6):609‐622. doi:10.1016/S1474-4422(13)70090-5 23684085

[6] Coyle‐Gilchrist IT , Dick KM , Patterson K , et al. Prevalence, characteristics, and survival of frontotemporal lobar degeneration syndromes. Neurology. 2016;86(18):1736‐1743. doi:10.1212/wnl.0000000000002638 27037234 PMC4854589

[7] Zhang Y , Wu KM , Yang L , Dong Q , Yu JT . Tauopathies: new perspectives and challenges. Mol Neurodegener. 2022;17(1):28. doi:10.1186/s13024-022-00533-z 35392986 PMC8991707

[8] Feldman HH , Cummings JL , Boxer AL , et al. A framework for translating tauopathy therapeutics: drug discovery to clinical trials. Alzheimers Dement. 2024;20(11):8129‐8152. doi:10.1002/alz.14250 39316411 PMC11567863

[9] Cummings JL , Gonzalez MI , Pritchard MC , May PC , Toledo‐Sherman LM , Harris GA . The therapeutic landscape of tauopathies: challenges and prospects. Alzheimers Res Ther. 2023;15(1):168. doi:10.1186/s13195-023-01321-7 37803386 PMC10557207

[10] Martínez‐Maldonado A , Ontiveros‐Torres MÁ , Harrington CR , et al. Molecular processing of tau protein in progressive supranuclear palsy: neuronal and glial degeneration. J Alzheimers Dis. 2021;79(4):1517‐1531. doi:10.3233/JAD-201139 33459640 PMC7990452

[11] Arakhamia T , Lee CE , Carlomagno Y , et al. Posttranslational modifications mediate the structural diversity of tauopathy strains. Cell. 2020;180(4):633‐644.e12. doi:10.1016/j.cell.2020.01.027 32032505 PMC7491959

[12] Karikari TK , Nagel DA , Grainger A , et al. Distinct conformations, aggregation and cellular internalization of different tau strains. Front Cell Neurosci. 2019;13:296. doi:10.3389/fncel.2019.00296 31338022 PMC6629824

[13] Kametani F , Yoshida M , Matsubara T , et al. Comparison of common and disease‐specific post‐translational modifications of pathological tau associated with a wide range of tauopathies. Front Neurosci. 2020;14:581936‐581936. doi:10.3389/fnins.2020.581936 33250706 PMC7672045

[14] Forrest SL , Kril JJ , Halliday GM . Cellular and regional vulnerability in frontotemporal tauopathies. Acta Neuropathol. 2019;138(5):705‐727. doi:10.1007/s00401-019-02035-7 31203391

[15] Panda D , Samuel Jonathan C , Massie M , Feinstein Stuart C , Wilson L . Differential regulation of microtubule dynamics by three‐ and four‐repeat tau: Implications for the onset of neurodegenerative disease. Proc Natl Acad Sci U S A. 2003;100(16):9548‐9553. doi:10.1073/pnas.1633508100 12886013 PMC170955

[16] Kyalu Ngoie Zola N , Balty C , Pyr dit Ruys S , et al. Specific post‐translational modifications of soluble tau protein distinguishes Alzheimer's disease and primary tauopathies. Nat Comm. 2023;14(1):3706. doi:10.1038/s41467-023-39328-1 PMC10287718 37349319

[17] Bronner IF , ter Meulen BC , Azmani A , et al. Hereditary Pick's disease with the G272V tau mutation shows predominant three‐repeat tau pathology. Brain. 2005;128(11):2645‐2653. doi:10.1093/brain/awh591 16014652

[18] Boxer AL , Geschwind MD , Belfor N , et al. Patterns of brain atrophy that differentiate corticobasal degeneration syndrome from progressive supranuclear palsy. Arch Neurol. 2006;63(1):81‐86. doi:10.1001/archneur.63.1.81 16401739

[19] Forman MS , Zhukareva V , Bergeron C , et al. Signature tau neuropathology in gray and white matter of corticobasal degeneration. Am J Pathol. 2002;160(6):2045‐2053. doi:10.1016/S0002-9440(10)61154-6 12057909 PMC1850831

[20] Kouri N , Whitwell JL , Josephs KA , Rademakers R , Dickson DW . Corticobasal degeneration: a pathologically distinct 4R tauopathy. Nat Rev Neurol. 2011;7(5):263‐272. doi:10.1038/nrneurol.2011.43 21487420 PMC10006729

[21] Farrell K , Humphrey J , Chang T , et al. Genetic, transcriptomic, histological, and biochemical analysis of progressive supranuclear palsy implicates glial activation and novel risk genes. Nat Comm. 2024;15(1):7880. doi:10.1038/s41467-024-52025-x PMC11385559 39251599

[22] Kovacs GG , Lukic MJ , Irwin DJ , et al. Distribution patterns of tau pathology in progressive supranuclear palsy. Acta Neuropathol. 2020;140(2):99‐119. doi:10.1007/s00401-020-02158-2 32383020 PMC7360645

[23] Dickson DW . Pick's disease: a modern approach. Brain Pathol. 1998;8(2):339‐54. doi:10.1111/j.1750-3639.1998.tb00158.x 9546291 PMC8098155

[24] Uchihara T , Tsuchiya K . Neuropathology of Pick body disease. Handb Clin Neurol. 2008;89:415‐430. 18631764 10.1016/S0072-9752(07)01238-9

[25] Yoshida M . Astrocytic inclusions in progressive supranuclear palsy and corticobasal degeneration. Neuropathology. 2014;34(6):555‐570. doi:10.1111/neup.12143 25124031

[26] Dickson DW , Bergeron C , Chin SS , et al. Office of Rare Diseases neuropathologic criteria for corticobasal degeneration. J Neuropathol Exp Neurol. 2002;61(11):935‐946. doi:10.1093/jnen/61.11.935 12430710

[27] Olfati N , Shoeibi A , Litvan I . Clinical spectrum of tauopathies. Front Neurol. 2022;13:944806. doi:10.3389/fneur.2022.944806 35911892 PMC9329580

[28] Ferrer I , López‐González I , Carmona M , et al. Glial and neuronal tau pathology in tauopathies: characterization of disease‐specific phenotypes and tau pathology progression. J Neuropathol Exp Neurol. 2014;73(1):81‐97. doi:10.1097/NEN.0000000000000030 24335532

[29] Goedert M , Jakes R . Mutations causing neurodegenerative tauopathies. Biochim Biophys Acta. 2005;1739(2):240‐250. doi:10.1016/j.bbadis.2004.08.007 15615642

[30] Sanchez‐Contreras MY , Kouri N , Cook CN , et al. Replication of progressive supranuclear palsy genome‐wide association study identifies SLCO1A2 and DUSP10 as new susceptibility loci. Mol Neurodegener. 2018;13(1):37. doi:10.1186/s13024-018-0267-3 29986742 PMC6038352

[31] Massimo L , Rennert L , Xie SX , et al. Common genetic variation is associated with longitudinal decline and network features in behavioral variant frontotemporal degeneration. Neurobiol Aging. 2021;108:16‐23. doi:10.1016/j.neurobiolaging.2021.07.018 34474300 PMC8616801

[32] Askenazi M , Kavanagh T , Pires G , Ueberheide B , Wisniewski T , Drummond E . Compilation of reported protein changes in the brain in Alzheimer's disease. Nat Comm. 2023;14(1):4466. doi:10.1038/s41467-023-40208-x PMC10368642 37491476

[33] Drummond E , Kavanagh T , Pires G , et al. The amyloid plaque proteome in early onset Alzheimer's disease and Down syndrome. Acta Neuropathol Commun. 2022;10(1):53. doi:10.1186/s40478-022-01356-1 35418158 PMC9008934

[34] Leitner D , Pires G , Kavanagh T , et al. Similar brain proteomic signatures in Alzheimer's disease and epilepsy. Acta Neuropathol. 2024;147(1):27. doi:10.1007/s00401-024-02683-4 38289539 PMC10827928

[35] Drummond E , Pires G , MacMurray C , et al. Phosphorylated tau interactome in the human Alzheimer's disease brain. Brain. 2020;143(9):2803‐2817. doi:10.1093/brain/awaa223 32812023 PMC7526722

[36] Swarup V , Chang TS , Duong DM , et al. Identification of conserved proteomic networks in neurodegenerative dementia. Cell Rep. 2020;31(12):107807. doi:10.1016/j.celrep.2020.107807 32579933 PMC8221021

[37] Dick F , Johanson GAS , Tysnes OB , Alves G , Dölle C , Tzoulis C . Brain proteome profiling reveals common and divergent signatures in parkinson's disease, multiple system atrophy, and progressive supranuclear palsy. Mol Neurobiol. 2025;62(3):2801‐2816. doi:10.1007/s12035-024-04422-y 39164482 PMC11790761

[38] Wojtas AM , Dammer EB , Guo Q , et al. Proteomic changes in the human cerebrovasculature in Alzheimer's disease and related tauopathies linked to peripheral biomarkers in plasma and cerebrospinal fluid. Alzheimers Dement. 2024;20(6):4043‐4065. doi:10.1002/alz.13821 38713744 PMC11180878

[39] Jang Y , Thuraisamy T , Redding‐Ochoa J , et al. Mass spectrometry‐based proteomics analysis of human globus pallidus from progressive supranuclear palsy patients discovers multiple disease pathways. Clin Transl Med. 2022;12(11):e1076. doi:10.1002/ctm2.1076 36354133 PMC9647849

[40] Lachén‐Montes M , González‐Morales A , Schvartz D , et al. The olfactory bulb proteotype differs across frontotemporal dementia spectrum. J Proteom. 2019;201:37‐47. doi:10.1016/j.jprot.2019.04.011 30999060

[41] Diner I , Nguyen T , Seyfried NT . Enrichment of detergent‐insoluble protein aggregates from human postmortem brain. J Vis Exp. 2017;(128):55835. doi:10.3791/55835 29155708 PMC5755167

[42] Hales CM , Dammer EB , Deng Q , et al. Changes in the detergent‐insoluble brain proteome linked to amyloid and tau in Alzheimer's disease progression. Proteomics. 2016;16(23):3042‐3053. doi:10.1002/pmic.201600057 27718298 PMC5462625

[43] Guo Q , Dammer EB , Zhou M , et al. Targeted quantification of detergent‐insoluble rna‐binding proteins in human brain reveals stage and disease specific co‐aggregation in Alzheimer's disease. Front Mol Neurosci. 2021;14:623659. doi:10.3389/fnmol.2021.623659 33815056 PMC8014091

[44] Mukherjee S , Dubois C , Perez K , et al. Quantitative proteomics of tau and Aβ in detergent fractions from Alzheimer's disease brains. J Neurochem. 2023;164(4):529‐552. doi:10.1111/jnc.15713 36271678

[45] Bache N , Geyer PE , Bekker‐Jensen DB , et al. A Novel LC system embeds analytes in pre‐formed gradients for rapid, ultra‐robust proteomics. Mol Cell Proteomics. 2018;17(11):2284‐2296. doi:10.1074/mcp.TIR118.000853 30104208 PMC6210218

[46] Ludwig C , Gillet L , Rosenberger G , Amon S , Collins BC , Aebersold R . Data‐independent acquisition‐based SWATH‐MS for quantitative proteomics: a tutorial. Mol Syst Biol. 2018;14(8):e8126. doi:10.15252/msb.20178126 30104418 PMC6088389

[47] Drummond E , Nayak S , Faustin A , et al. Proteomic differences in amyloid plaques in rapidly progressive and sporadic Alzheimer's disease. Acta Neuropathol. 2017;133(6):933‐954. doi:10.1007/s00401-017-1691-0 28258398 PMC5503748

[48] Kavanagh T , Balcomb K , Ahmadi Rastegar D , et al. hnRNP A1, hnRNP A2B1, and hnRNP K are dysregulated in tauopathies, but do not colocalize with tau pathology. Brain Pathol. 2025;35:e13305. doi:10.1111/bpa.13305 39354671 PMC11961206

[49] Afroz T , Skrisovska L , Belloc E , Guillén‐Boixet J , Méndez R , Allain FH . A fly trap mechanism provides sequence‐specific RNA recognition by CPEB proteins. Genes Dev. 2014;28(13):1498‐514. doi:10.1101/gad.241133.114 24990967 PMC4083092

[50] Michetti F , D'Ambrosi N , Toesca A , et al. The S100B story: from biomarker to active factor in neural injury. J Neurochem. 2019;148(2):168‐187. doi:10.1111/jnc.14574 30144068

[51] Hu Y‐B , Zou Y , Huang Y , et al. ROCK1 is associated with Alzheimer's disease‐specific plaques, as well as enhances autophagosome formation but not autophagic aβ clearance. Front Cell Neurosci . 2016;10:253. doi:10.3389/fncel.2016.00253 27853422 PMC5089992

[52] Gurkar AU , Chu K , Raj L , et al. Identification of ROCK1 kinase as a critical regulator of Beclin1‐mediated autophagy during metabolic stress. Nat Comm. 2013;4(1):2189. doi:10.1038/ncomms3189 PMC3740589 23877263

[53] Huang H , Kong D , Byun KH , et al. Rho‐kinase regulates energy balance by targeting hypothalamic leptin receptor signaling. Nat Neurosci. 2012;15(10):1391‐1398. doi:10.1038/nn.3207 22941110 PMC3458121

[54] Misawa H , Kawasaki Y , Mellor J , et al. Contrasting localizations of MALS/LIN‐7 PDZ proteins in brain and molecular compensation in knockout mice*. JBC. 2001;276(12):9264‐9272. doi:10.1074/jbc.M009334200 11104771

[55] Wang Y , Lyu L , Vu T , McCarty N . TRIM44 enhances autophagy via SQSTM1 oligomerization in response to oxidative stress. Sci Rep. 2024;14(1):18974. doi:10.1038/s41598-024-67832-x 39152142 PMC11329658

[56] Wallace EW , Kear‐Scott JL , Pilipenko EV , et al. Reversible, specific, active aggregates of endogenous proteins assemble upon heat stress. Cell. 2015;162(6):1286‐98. doi:10.1016/j.cell.2015.08.041 26359986 PMC4567705

[57] Banani SF , Lee HO , Hyman AA , Rosen MK . Biomolecular condensates: organizers of cellular biochemistry. Nat Rev Mol Cell Biol. 2017;18(5):285‐298. doi:10.1038/nrm.2017.7 28225081 PMC7434221

[58] Markmiller S , Soltanieh S , Server KL , et al. Context‐dependent and disease‐specific diversity in protein interactions within stress granules. Cell. 2018;172(3):590‐604.e13. doi:10.1016/j.cell.2017.12.032 29373831 PMC5969999

[59] Sui X , Pires DEV , Ormsby AR , et al. Widespread remodeling of proteome solubility in response to different protein homeostasis stresses. Proc Natl Acad Sci U S A. 2020;117(5):2422‐2431. doi:10.1073/pnas.1912897117 31964829 PMC7007570

[60] Apicco DJ , Ash PEA , Maziuk B , et al. Reducing the RNA binding protein TIA1 protects against tau‐mediated neurodegeneration in vivo. Nat Neurosci. 2018;21(1):72‐80. doi:10.1038/s41593-017-0022-z 29273772 PMC5745051

[61] Lantero‐Rodriguez J , Camporesi E , Montoliu‐Gaya L , et al. Tau protein profiling in tauopathies: a human brain study. Mol Neurodegener. 2024;19(1):54. doi:10.1186/s13024-024-00741-9 39026372 PMC11264707

[62] Stanford PM , Shepherd CE , Halliday GM , et al. Mutations in the tau gene that cause an increase in three repeat tau and frontotemporal dementia. Brain. 2003;126(4):814‐826. doi:10.1093/brain/awg090 12615641

[63] Jiang Z , Cote J , Kwon Jennifer M , Goate Alison M , Wu Jane Y . Aberrant splicing of tau pre‐mRNA caused by intronic mutations associated with the inherited dementia frontotemporal dementia with parkinsonism linked to chromosome 17. Mol Cell Biol. 2000;20(11):4036‐4048. doi:10.1128/MCB.20.11.4036-4048.2000 10805746 PMC85774

[64] Zareba‐Paslawska J , Patra K , Kluzer L , Revesz T , Svenningsson P . Tau isoform‐driven CBD pathology transmission in oligodendrocytes in humanized tau mice. Front Neurol. 2021;11(1825):589471. doi:10.3389/fneur.2020.589471 33519674 PMC7845573

[65] He Z , McBride JD , Xu H , et al. Transmission of tauopathy strains is independent of their isoform composition. Nat Comm. 2020;11(1):7. doi:10.1038/s41467-019-13787-x PMC6946697 31911587

[66] Chen D , Drombosky KW , Hou Z , et al. Tau local structure shields an amyloid‐forming motif and controls aggregation propensity. Nat Comm. 2019;10(1):2493. doi:10.1038/s41467-019-10355-1 PMC6555816 31175300

[67] Sandhir R , Onyszchuk G , Berman NE . Exacerbated glial response in the aged mouse hippocampus following controlled cortical impact injury. Exp Neurol. 2008;213(2):372‐80. doi:10.1016/j.expneurol.2008.06.013 18692046 PMC2662478

[68] Gerlach R , Demel G , König HG , et al. Active secretion of S100B from astrocytes during metabolic stress. Neuroscience. 2006;141(4):1697‐1701. doi:10.1016/j.neuroscience.2006.05.008 16782283

[69] Sanyal A , DeAndrade MP , Novis HS , et al. Lysosome and inflammatory defects in GBA1‐mutant astrocytes are normalized by LRRK2 inhibition. Mov Disord. 2020;35(5):760‐773. doi:10.1002/mds.27994 32034799 PMC8167931

[70] Miyakawa S , Sakuma H , Warude D , et al. Anti‐sortilin1 antibody up‐regulates progranulin via sortilin1 down‐regulation. Front Neurosci. 2020;14:586107. doi:10.3389/fnins.2020.586107 33384578 PMC7770147

[71] Prudencio M , Jansen‐West KR , Lee WC , et al. Misregulation of human sortilin splicing leads to the generation of a nonfunctional progranulin receptor. Proc Natl Acad Sci U S A. 2012;109(52):21510‐21515. doi:10.1073/pnas.1211577110 23236149 PMC3535614

[72] Takahashi H , Bhagwagar S , Nies SH , et al. Reduced progranulin increases tau and α‐synuclein inclusions and alters mouse tauopathy phenotypes via glucocerebrosidase. Nat Comm. 2024;15(1):1434. doi:10.1038/s41467-024-45692-3 PMC10873339 38365772

[73] Jiang J , Yang C , Ai JQ , et al. Intraneuronal sortilin aggregation relative to granulovacuolar degeneration, tau pathogenesis and sorfra plaque formation in human hippocampal formation. Front Aging Neurosci. 2022;14:926904. doi:10.3389/fnagi.2022.926904 35978952 PMC9376392

[74] Hu X , Hu ZL , Li Z , et al. Sortilin fragments deposit at senile plaques in human cerebrum. Front Neuroanat. 2017;11:45. doi:10.3389/fnana.2017.00045 28638323 PMC5461299

[75] Tu T , Jiang J , Zhang QL , et al. Extracellular sortilin proteopathy relative to β‐amyloid and tau in aged and Alzheimer's disease human brains. Front Aging Neurosci. 2020;12:93. doi:10.3389/fnagi.2020.00093 32477092 PMC7236809

[76] Kurnellas M , Mitra A , Schwabe T , et al. Latozinemab, a novel progranulin‐elevating therapy for frontotemporal dementia. J Transl Med 2023;21(1):387. doi:10.1186/s12967-023-04251-y 37322482 PMC10268535

[77] Chen W , Nyuydzefe MS , Weiss JM , Zhang J , Waksal SD , Zanin‐Zhorov A . ROCK2, but not ROCK1 interacts with phosphorylated STAT3 and co‐occupies TH17/TFH gene promoters in TH17‐activated human T cells. Sci Rep. 2018;8(1):16636. doi:10.1038/s41598-018-35109-9 30413785 PMC6226480

[78] Sogorb‐Esteve A , Weiner S , Simrén J , et al. Proteomic analysis reveals distinct cerebrospinal fluid signatures across genetic frontotemporal dementia subtypes. Sci Transl Med. 2025;17(784):eadm9654. doi:10.1126/scitranslmed.adm9654 39908349

