# Supplementary material for: Differences in the soluble and insoluble proteome between primary tauopathies
Source: Alzheimers Dement. 2025 Jun 22;21(6):e70401. doi: 10.1002/alz.70401 (PMC12183113; doi:10.1002/alz.70401)
Supplement: Supplementary file 2 — Supporting Information [file ALZ-21-e70401-s003.pdf]

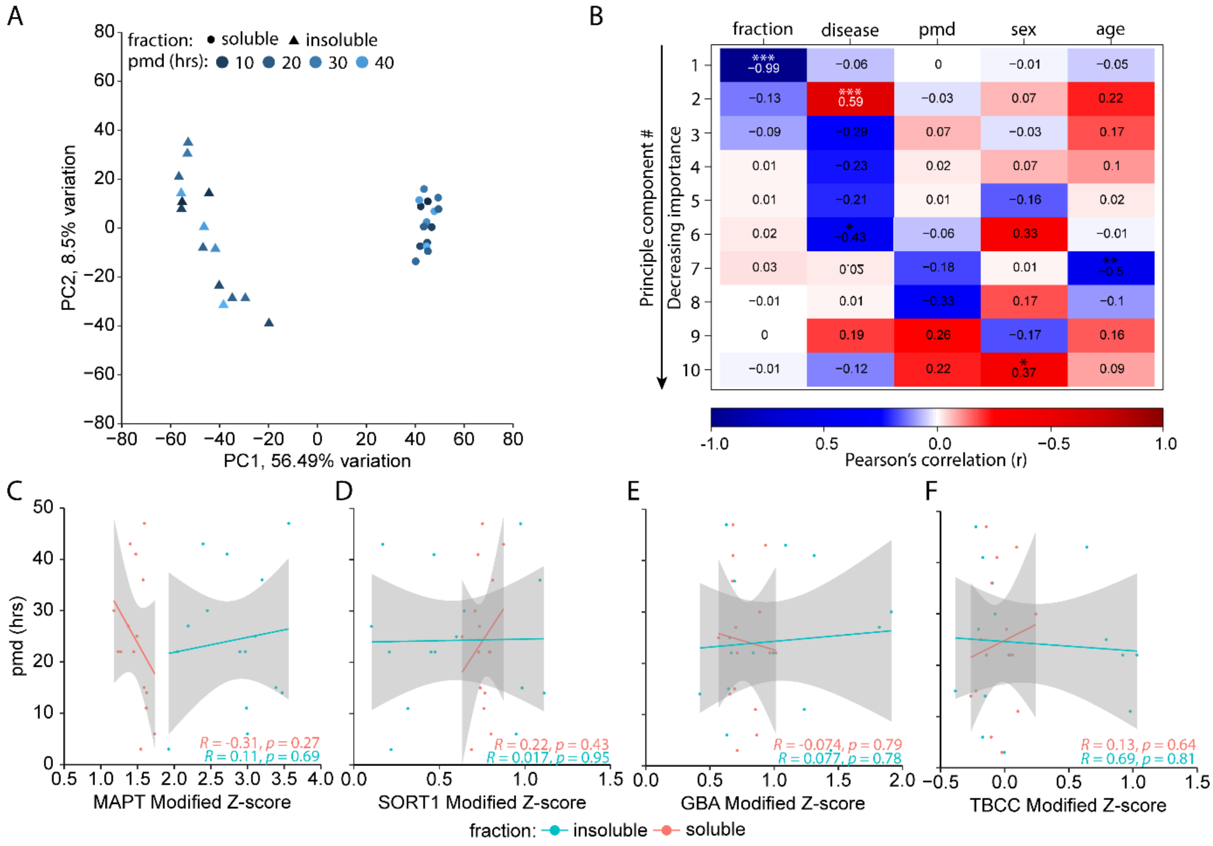

Supplementary Figure 1: Assessment of covariate influence on proteomics data

A PCA plot of raw proteomics data colored for PMD (hrs). **B** Correlogram of PCA eigen value correlations with each co-variate. **C-F** Correlation between pmd (hrs) and modified z-scores for soluble and insoluble fractions of proteins of interest **C** MAPT, **D** SORT1, **E** GBA and **F** TBCC.

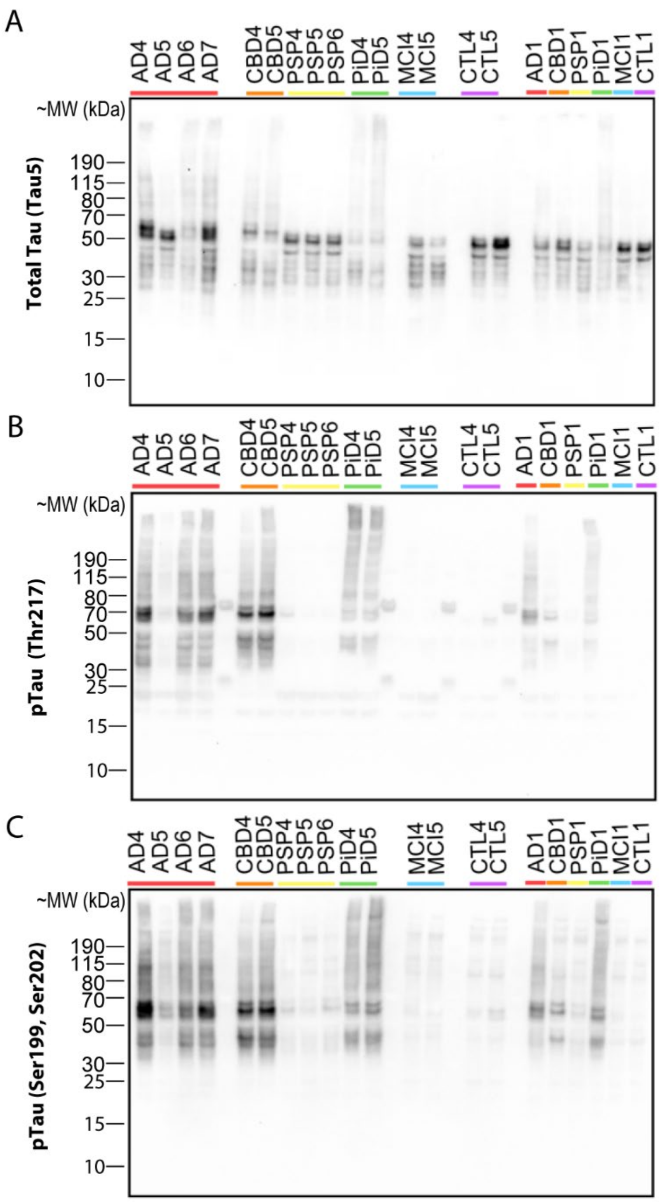

Supplementary Figure 2: Western blots of total homogenate for tau epitopes

Western blots of the remaining two cases not shown in Figure 1, probed for **A** total tau (Tau-5) **B** pT217 tau and **C** pS199/pS202 tau.

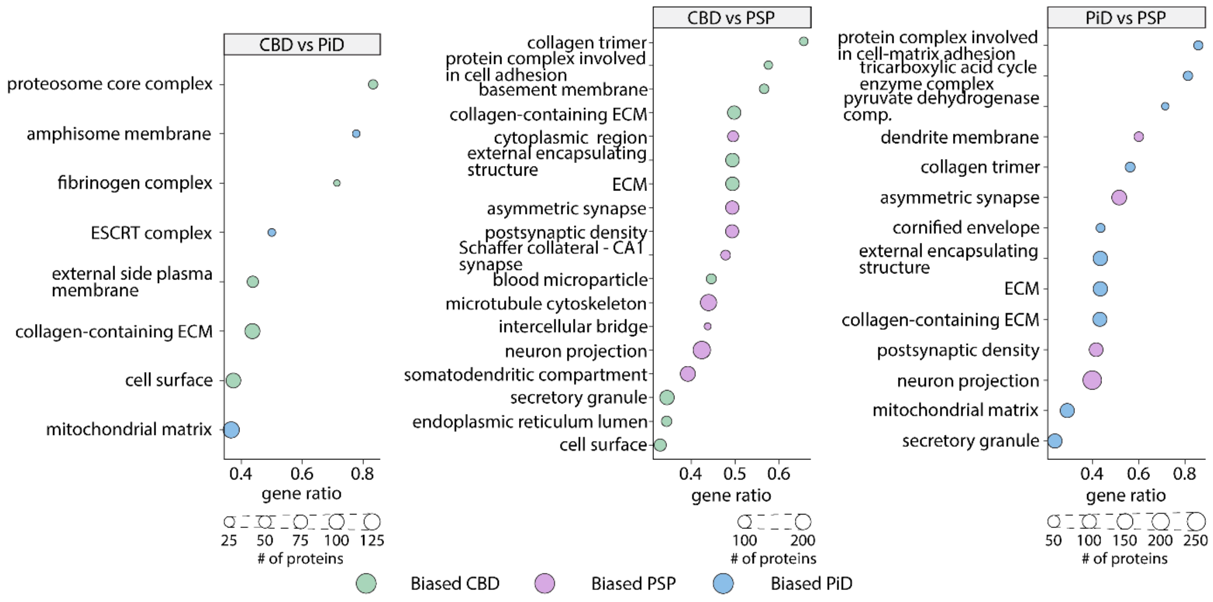

Supplementary Figure 3: Gene set enrichment analysis for protein solubility differences

Top 10 GO cellular compartments identified by GSEA for CBD vs PiD, CBD vs PSP and PiD vs PSP complex statistical comparisons (Figure 1B, iii)
